# Diagnostic accuracy of the clinical indicators to identify central sensitization pain in patients with musculoskeletal pain

**DOI:** 10.1186/s40945-020-00095-7

**Published:** 2021-01-11

**Authors:** Juliana Valentim Bittencourt, Ana Carolina de Melo Magalhães Amaral, Pedro Vidinha Rodrigues, Leticia Amaral Corrêa, Bruno Moreira Silva, Felipe José Jandre Reis, Leandro Alberto Calazans Nogueira

**Affiliations:** 1 Rehabilitation Science Postgraduate Program at Augusto Motta University Centre (UNISUAM), Avenida Paris, 84, Bonsucesso, Rio de Janeiro, RJ CEP 21041-020 Brazil; 2 Physiotherapy Department at Federal Institute of Rio de Janeiro (IFRJ), Rio de Janeiro, Brazil; 3 Department of Physiology, Federal University of São Paulo, São Paulo, SP Brazil

**Keywords:** Musculoskeletal pain, Chronic pain, Pain mechanisms, Central nervous system sensitization, Diffuse noxious inhibitory control, Pain threshold, Pain management

## Abstract

**Background:**

The identification of central sensitization (CS) is an important aspect in the management of patients with chronic musculoskeletal pain. Several methods have been developed, including clinical indicators and psychophysical measures. However, whether clinical indicators coincide with the psychophysical test of CS-related sign and symptoms is still unknown. Therefore, the present study aimed to analyze the diagnostic accuracy of the clinical indicators in identifying CS-related sign and symptoms in patients with musculoskeletal pain.

**Methods:**

One-hundred consecutive patients with musculoskeletal pain were included. Clinical indicators (index method) based on a combination of patient self-report pain characteristics and physical examination were used to identify the phenotype of patients with musculoskeletal pain and the predominance of the CS-related sign and symptoms. Conditioned pain modulation (CPM) was assessed by the Cold Pressor Test (reference standard), which is a psychophysical test used to detect impairment of CPM. Measurements of the diagnostic accuracy were performed.

**Results:**

Twenty-seven patients presented predominance of CS-related sign and symptoms in the assessment of the clinical indicators, and 20 had impairment of CPM. Clinical indicators showed high accuracy (75.0%; 95% confidence interval = 65.3 to 83.1), high specificity (80.0%; 95% confidence interval = 69.6 to 88.1), high negative predictive value (87.7%; 95% confidence interval = 81.2 to 92.1), and a relevant positive likelihood ratio (2.8, 95% confidence interval = 1.5 to 5.0) when compared to the Cold Pressor Test.

**Conclusion:**

Clinical indicators demonstrated a valuable tool for detecting the impaired CPM, which is a remarkable feature of the CS-related sign and symptoms. Clinicians are encouraged to use the clinical indicators in the management of patients with musculoskeletal pain.

## Background

Musculoskeletal health conditions are a common cause of pain in the general population, and the central sensitization (CS) is linked with a number of these patients. Musculoskeletal pain is present in approximately half of the population of Europe [1], the United States [2], and Brazil [3]. People with musculoskeletal health conditions may develop a chronic pain condition that affects approximately 40% of the world’s population [4]. A study evidenced that 41% of Brazilian people have pain with more than 6 months of duration [5]. The persistent feature of pain has been associated with the CS. Several studies in patients with musculoskeletal pain revealed the implication of the CS in the perception of pain in patients with musculoskeletal conditions [6–9]. A narrative review revealed that these conditions were categorized as psychosomatic, functional, somatization, and medically unexplained disorders [10]. Previously, Yunus proposed the term “CS syndrome” for non-organic disorders that share several common characteristics, including pain and psychosocial factors [11].

Identifying the pain related to CS is a clinical challenge for health professionals because there is no gold standard assessment method [12]. Besides, the definition of CS remains uncertain. According to Woolf, CS is defined as the “amplification of neural signaling within the central nervous system that elicits pain hypersensitivity” [6]. Other authors argue that CS encompasses impaired conditioned pain modulation (CPM) [13], and activation of descending and ascending facilitatory pain pathways [14]. Some instruments are available to identify musculoskeletal pain patients related to the CS, being the CPM, most used for this purpose. CPM occurs when a conditioning stimulus [i.e., cold pressor test (CPT) involving the immersion of the hand in cold water] inhibited a painful stimulus. Although CPT has been the most commonly used method for the evaluation of CPM [15], this psychophysical test can be impractical for routine clinical screening because of the requirement of specific apparatus (i.e., thermometer, a container with ice and pressure algometer). On the other hand, clinical indicators encompass a group of information gathered during regular clinical evaluation, albeit whether CPM is impaired in patients with the clinical phenotype of CS remains unclear.

The relevant frequency of the patients with chronic musculoskeletal pain and the predominance of the CS phenotype [16] led researchers to propose tools for identifying this clinical phenotype [7, 17–19]. Health care professionals developed a consensus-derived list of signs and symptoms (clinical indicators) suggestive for each pain mechanism (nociceptive, peripheral neuropathic or CS) [20]. Hence, clinical indicators based on a combination of patient self-report pain characteristics and physical examination were described to identify the mechanism of musculoskeletal pain [20]. Previous study confirmed the discriminative validity for the identification of each pain dominance in patients with low back pain [19]. Clinical indicators showed high reliability in patients with low back pain [21] and patients with nonspecific neck pain [22]. Besides, clinical indicators have been recommended for the management of low back pain [9], nonspecific shoulder pain [23], and chronic pain related to osteoarthritis [24]. Although the clinical indicators are based on the perspectives of clinicians, our group found similar prevalence of patients with CS-related sign and symptoms (21%) [16] and impaired CPM (25%) [25] in patients with musculoskeletal pain. Accordingly, there is a need to verify whether the clinical indicators are accurate to detect impairment of CPM.

The lack of robust terminology to identify clinical features and neurophysiological mechanisms hamper definite decision-making in patients with chronic musculoskeletal pain. Clinical indicators have the potential to be a suitable tool for screening purposes and helpful for guiding the management of patients with different pain. Still, an investigation of the criterion validity of the clinical indicators (index method) to detect the impairment of CPM using a psychophysical test (reference standard) is needed to ensure an adequate classification of the patients. Therefore, the present study aimed to analyze the diagnostic accuracy of the clinical indicators in identifying impairment of CPM in patients with musculoskeletal pain. We hypothesized that patients with musculoskeletal pain with the clinical phenotype of the CS-related sign and symptoms would present impairment of the CPM.

## Methods

### Study design and ethical considerations

This diagnostic accuracy study followed the Standards for Reporting Studies of Diagnostic Accuracy (STARD) [26]. The study was approved by the Research Ethics Committee of Augusto Motta University Centre (CAAE number: 46245215.9.0000.5235), following the Helsinki Declaration for research in humans. All patients who met the eligibility criteria signed the informed consent form before the study procedures.

### Study patients

Consecutive patients with musculoskeletal pain (aged 18 years and over) of the outpatient physiotherapy of Gaffrée and Guinle University Hospital were enrolled when they sought treatment between November 2015 and May 2016. The study included patients with acute pain (pain duration less than 3 months) and chronic pain (pain duration greater than 3 months). Musculoskeletal pain was defined as pain perceived in a region of the body with muscular, ligament, bone, or joint origin [2]. The study excluded patients who had a surgical procedure in the spine, pregnant women, patients with rheumatologic diagnosis in the acute inflammatory phase, tumors, being illiterate, or could not complete the self-reported questionnaires.

### Procedures

Patients were referred for an initial evaluation consisting of the clinical history and physical examination. The acquisition of sociodemographic and clinical information was performed by an instrument containing demographic data (full name, sex, age, address, educational level, occupational, marital status), characteristics of musculoskeletal pain (pain location, pain intensity, pain duration), and physical exercise behavior. Participants completed items regarding clinical indicators for the predominance of the musculoskeletal pain based on its mechanism [19], which classify the patients into nociceptive, peripheral neuropathic or CS-related sign and symptoms. Then, the participants were instructed to perform the psychophysical test, which was conducted on the same day.

Pain intensity was measured using the Numeric Pain Rating Scale (NPRS) from 0 to 10 (i.e., 0 is no pain, and 10 is the worst pain possible). The duration of pain was recorded in months, and patients were classified with chronic musculoskeletal pain if they had pain for more than 3 months, according to the International Association for the Study of Pain definition [27]. The physical exercise behavior was self-reported, and it was defined as a form of physical activity that is planned, structured, repetitive, and aims to improve or maintain physical fitness [28]. The completion of the questionnaires lasted approximately 10 min per participant and was supervised by an examiner for clarification in case of uncertainties.

### Measuring instruments

#### Clinical phenotype of CS

The classification of musculoskeletal pain in nociceptive, peripheral neuropathic pain, and CS-related sign and symptoms were identified based on the recognition of clinical indicators, which uses a combination of patient self-report pain characteristics and physical examination. The physical examination included musculoskeletal and neurological-based assessments. Two physiotherapists performed the predominance of musculoskeletal pain classification. The two physiotherapists involved (L.A.C.N. and F.J.J.R.) had 16 years of work experience in an outpatient department in treating patients with musculoskeletal disorders. A standard procedure was defined by one examiner (L.A.C.N) who also delivered a 3-h assessment protocol training session to the other (F.J.J.R.) in order to clarify and confirm understanding of the assessment procedure. Any doubts that arose during this process were resolved by reaching a consensus between the two investigators. The following indicators defined the predominance of each musculoskeletal pain.

Central sensitization: A dominance of CS-related sign and symptoms was considered using a four-criteria cluster as described early in the literature [19]: (1) pain disproportionate to the nature and extent of injury or pathology; (2) a disproportionate, non-mechanical, unpredictable pattern of pain provocation in response to multiple/non-specific aggravating/easing factors; (3) a strong association with maladaptive psychosocial factors, and (4) one sign (diffuse/non-anatomical areas of pain/tenderness on palpation). The four-criteria cluster showed a sensitivity of 91.8% and a specificity of 97.7% compared to an experienced clinical judgment [19].

Nociceptive pain: The seven-criteria cluster included the presence/absence symptoms. The presence of (1) usually intermittent and sharp with movement/mechanical provocation; may be a more constant dull ache or throb at rest; (2) pain localized to the area of injury/dysfunction; (3) clear, proportionate mechanical/anatomical nature to aggravating and easing factors). The absence of (1) pain variously described as burning, shooting, sharp or electric shock-like, (2) pain in association with other dysesthesias, (3) night pain/disturbed sleep), and (4) one sign (antalgic postures/movement patterns) [19]. A dominance of nociceptive pain was predicted by a seven-criteria cluster with a sensitivity of 90.9% and a specificity of 91.0% compared to an experienced clinical judgment [19].

Peripheral neuropathic pain: The three-criteria cluster included the presence of three characteristics: (1) history of nerve injury, pathology or mechanical compromise; (2) pain referred to in a dermatomal, or cutaneous distribution, and (3) pain/symptom provocation with mechanical/movement tests). A neurological physical assessment was based on a classical neurological examination, which was done to confirm the peripheral neuropathic pain, and then the dermatomal distribution of pain. Muscle weakness tests, neurodynamic tests (i.e., slump, sciatic, femoral, median, ulnar and radial), and testing of the function of the sensory fibers were conducted to confirm hypothesis [19]. A dominance of peripheral neuropathic pain was found with a three-criteria cluster with a sensitivity of 86.3% and a specificity of 96.0% compared to an experienced clinical judgment [19].

#### Neurophysiological feature of CS

Conditioned Pain Modulation – The Cold Pressor Test (CPT) was the psychophysical test used to measure the CPM. The CPT uses the conditioning stimulus of pain to measure the CPM, which is an appropriate method to assess the descending nociceptive inhibitory system [5]. The conditioning stimulus was the immersion of the participants’ non-dominant and asymptomatic hand in a bucket with temperature-controlled cold water (1 °C – 4 °C) monitored by a thermometer (5130 model, Incoterm), for up to 1 min. The participant was instructed to remain with the hand immersed in water without making muscle contractions or changes in position. The withdrawal of the side from the water was allowed when the patient could no longer tolerate the painful stimulus. Room temperature, humidity, lighting, and noise were maintained constant during the entire procedure.

The pressure pain threshold (PPT) was performed in the forearm regions and tibialis anterior muscle of the dominant limbs before and after 1 min of the CPT, using a digital pressure algometer (model Force Ten FDX, Wagner Instruments, Greenwich, USA). Tibialis anterior muscle and the distal part of the dorsal forearm, which had not been immersed in water, were chosen to be evaluated due to the lack of relationship with participants` musculoskeletal complaints. The operation of the pressure algometer and measurement of PPT were explained to patients before the assessment. Besides, a familiarization procedure was carried out with the pressure algometer by applying pressure to the dominant forearm to ensure that the test had been understood. The force was gradually increased (1 kg-force/s) until the feeling of pressure from the primary subject was changed to pain. The PPT was recorded in kilograms-force (Kgf) when the patient gave the verbal command “pain.”

Only patients with the inefficiency of the CPM in both locations (the anterior tibialis muscle and the distal part of the dorsal forearm) were classified as impaired CPM. Upper and lower limb sites were used to avoid the inclusion of the patients with peripheral sensitization according to recent recommendations for CPM [29]. Also, the efficiency of the descending nociceptive inhibitory system was assessed by calculating the difference between the PPT values in CPT (final cost – initial value). Negative values represented an inefficiency of the descending nociceptive inhibitory system, and null or positive values were considered a typical response of the descending nociceptive inhibitory system.

### Statistical analysis

The demographic and clinical variables of the study population were presented as mean and standard deviation for continuous variables. Categorical variables were presented as absolute values and frequencies. The Shapiro-Wilk test verified the normal distribution of the majority of the continuous variables. We compared the group of patients who presented impairment of CPM with those with no impairment of CPM. The diagnostic accuracy of the clinical indicators (index method) was compared with the psychophysical measure (reference standard). We calculated sensitivity, specificity, positive predictive value, negative predictive value, diagnostic accuracy, positive likelihood ratio, and negative likelihood ratio were calculated. Results are presented with the respective 95% confidence interval (95% CI). A significance level of less than 5% (*P* < 0.05) was considered for all analyses. The statistical analysis was performed using SPSS version 20.0 (IBM Corporation, Armonk, New York).

## Results

### Characteristics of the participants

The study was composed of 100 patients with musculoskeletal pain being 27 males and 73 females with a mean age of 50.9 (±16.6) years old. Twelve (10.08%) participants were classified with acute pain and 88 (73.95%) with chronic pain. The mean weight was 72.9 (±15.4) kg, and the mean body mass was 25.9 (±5.2) kg/m^2^. Regarding pain characteristic, the mean pain intensity was 6.0 (±2.5) and the mean pain duration was 43.0 (±53.0) months. All participants completed the classification of the CS-related sign and symptoms using the clinical indicators and the CPT test. Then, there were no missing values for both classifications. There were no adverse events associated with the questionnaires and the psychophysical test.

### Identification of the CS

From the total, 27 participants presented the clinical phenotype of CS-related sign and symptoms, while 20 presented the impairment of the CPM, 16 of whom (80%) were women. Fourteen (14%) patients were classified as acute musculoskeletal pain and 86 (86%) as chronic musculoskeletal pain. The impairment of the CPM was observed in 4 (29%) patients with acute musculoskeletal pain and 16 (19%) patients with chronic musculoskeletal pain. A chi-square test revealed similar proportions of impairment of CPM between the groups (X^2^ = 0.748; *p* = 0.387). There were no significant differences in clinical and demographic characteristics between the two groups, and the data are shown in Table 1.

**Table 1:** Clinical and demographic characteristics of participants of the study (*n* = 100)

Characteristics	Impaired CPM (***n*** = 20)	Normal CPM (***n*** = 80)	***P*** value
Age, mean (SD)	55.3 (16.67)	49.8 (16.6)	0.195
Sex, n (%), female	16 (80%)	57 (71%)	0.430
Body mass index, mean (SD)	25.4 (3.17)	26.1 (3.17)	0.713
Physical Exercise (Yes), n (%)	5 (25%)	39 (48%)	0.056
Comorbidities, mean (DP)	0.9 (0.90)	0.8 (1.1)	0.824
Pain duration (months), mean (SD)	41.1 (48.5)	43.5 (54.4)	0.855
Pain Intensity, mean (SD)	7.0 (2.7)	5.8 (2.4)	0.090
Pain Location, n (%)			0.603
Neck Pain	–	5 (6%)	
Thoracic Pain	–	–	
Low Back Pain	1 (5%)	13 (16%)	
Head Pain	–	1 (1%)	
Upper Limb Pain	3 (15%)	7 (8%)	
Lower Limb Pain	3 (15%)	12 (15%)	
More than one location	9 (45%)	35 (43%)	
Pain phenotype, n (%)			0.001
Central Sensitization	11 (55%)	16 (20%)	
Nociceptive	8 (40%)	31 (39%)	
Peripheral Neuropathic	1 (5%)	33 (41%)	

Note: Data are presented as mean (SD) for continuous variables and as frequency counts (%) for categorical variables. Significant differences between groups were tested using the unpaired t-test for continuous variables or the Chi-square test for categorical variables

Abbreviations: *CPM* Conditioned pain modulation

Table 2 presents pressure pain threshold values for the dorsal region of the forearm and anterior tibial of the participants. The pain threshold at the dorsal forearm pressure was reduced in the participants with impairment of the CPM in the post-test evaluation [impaired CPM = 2.6 (±0.78), normal CPM = 5.4 (±2.5); *p* < 0.001)], as well as in the anterior tibial region [(impaired CPM = 4.0 (±2.2), normal CPM = 6.7 (±3.4); *p* < 0.001)]. Consequently, the within-group comparison was also statistically significant (Table 2).

**Table 2:** Pressure pain threshold values for the dorsal forearm and anterior tibial regions of the patients with musculoskeletal disorders (*n* = 100)

Characteristics	Impaired CPM (***n*** = 20)	Normal CPM (***n*** = 80)	***P***-value
Baseline			
Dorsal forearm algometry (kgf)	3.0 (0.8)	3.5 (1.8)	0.189
Tibialis anterior algometry (kgf)	5.0 (2.8)	5.1 (2.3)	0.792
After Cold Pressor Test			
Dorsal forearm algometry (kgf)	2.6 (0.8)	5.4 (2.5)	< 0.001*
Tibialis anterior algometry (kgf)	4.0 (2.2)	6.7 (3.4)	< 0.001*
Within-group change			
Dorsal forearm algometry (kgf)	−0.3 (0.5)	1.8 (1.3)	< 0.001*
Tibialis anterior algometry (kgf)	−0.9 (1.6)	1.5 (2.2)	< 0.001*

Note: Data are presented as mean (SD). Significant differences between groups were tested using the unpaired t-test. *Represents significant *P*-values (*P* < 0.05)

Abbreviations: *CPM* Conditioned pain modulation

### Diagnostic accuracy of the clinical indicators

Clinical indicators showed high specificity (80.0%; 95% CI 69.6 to 88.1), high accuracy (75.0%; 95% CI = 65.3 to 83.1), high negative predictive value (87.7%; 95% CI = 81.2 to 92.1) and a relevant positive likelihood ratio (2.8, 95% CI = 1.5 to 5.0), but low values of sensitivity (55.0%; 95% CI = 31.5 to 76.9) and positive predictive value (40.7; 95% CI = 27.6 to 55.4) when compared to the CPT. Measurements of sensitivity, specificity, positive predictive value, negative predictive value, and accuracy for the diagnosis of central sensitization are presented in Table 3.

**Table 3:** Values of sensitivity, specificity, positive predictive value and negative predictive value, accuracy, disease prevalence, and likelihood ratio (positive and negative) of the clinical diagnosis of CS-related sign and symptoms

	Clinical Diagnosis
Sensitivity %, (95% CI)	55.0 (31.5 to 76.9)
Specificity %, (95% CI)	80.0 (69.6 to 88.1)
Positive Predictive Value (PPV) %, (95% CI)	40.7 (27.6 to 55.4)
Negative Predictive Value (NPV) %, (95% CI)	87.7 (81.2 to 92.1)
Accuracy %, (95% CI)	75.0 (65.3 to 83.1)
Disease prevalence %, (95% CI)	20.0 (12.7 to 29.2)
Positive Likelihood Ratio (LR+) (95% CI)	2.8 (1.5 to 5.0)
Negative Likelihood Ratio (LR-) (95% CI)	0.6 (0.3 to 0.9)

Note: Abbreviation: *CI* Confidence interval, *PPV* Positive predictive value, *NPV* negative predictive value, *LR+* positive likelihood ratio, *LR-* Negative likelihood ratio

## Discussion

This study investigated the diagnostic accuracy of the clinical indicators in identifying patients with musculoskeletal pain and CS-related sign and symptoms, considering the impairment of the CPM as a standard measured. Clinical indicators exhibited high values of diagnostic accuracy, specificity, the negative predictive value of the clinical diagnosis of impairment of CPM in patients with musculoskeletal pain. Besides, the positive likelihood ratio found in the current study represents a relevant increase in odds favoring a rule in patients with the impairment of the CPM. The clinical indicators demonstrated to be useful to detect impairment of CPM in patients with musculoskeletal pain, specially ruled out those patients who do not have impairment of CPM.

We acknowledge strengths and limitations in the current study. The first strength is the novelty to validate the use of a practical and straightforward system of clinical identification of the phenotype of patients with CS-related sign and symptoms. The second strength was the use of a psychophysical method for impairment of CPM identification using two different anatomical sites for its classification. Ultimately, we enrolled target-positive and target-negative patients (i.e., patients with musculoskeletal pain with and without CS-related sign and symptoms from the same population in a consecutive patient sampling. Regarding the limitations of the study, CPT is not the gold-standard for the identification of the impairment of CPM. An experiment with secondary hyperalgesia induced by intradermal capsaicin injection was claimed for the confirmation of the CS [30]. Nevertheless, CPT is the most common method used for conditioned pain modulation assessment [15], which is an appropriate method to assess the descending nociceptive inhibitory system [31] and a component of CS. The number of participants enrolled in the current study may be insufficient for validation purposes, and a larger sample would be needed. Nonetheless, a total sample size equal to or greater than 100 participants have been considered an aspect for a strong level of evidence in studies of measurement properties [32–34]. The validation studies are representative of the sample studied, and our results must be tested in different populations to generalisability of the findings. Ultimately, we did not control the use of analgesic medication, which may affect the CPM response despite the contradictory results described in a systematic review [35].

Despite the high proportion of the participants without CS-related sign and symptoms in the clinical indicators were correctly diagnosed (i.e., negative predictive value), the negative likelihood ratio was not small enough to rule out the CS-related sign and symptoms with confidence. Predictive values provide probabilities of abnormality for a particular test, but the prevalence of the abnormality in the study sample interferes with the results. In the current study, the prevalence of the CS-related sign and symptoms was 27%, corroborating a previous Brazilian study [16] and other international studies [36]. Accordingly, the relatively low prevalence of the CS-related sign and symptoms in the pretest probability influenced the results of the likelihood ratio, which generated a small but important change in the posttest probability of the clinical identification of the CS-related sign and symptoms. Our findings revealed that patients with the impaired CPM were 2.8 times more likely to have CS-related sign and symptoms in the clinical indicators than the patients with preserved CPM. The notable finding of the positive likelihood ratio represents that the clinical indicators are useful to rule in patients with CS-related sign and symptoms. Therefore, the clinical indicators represent a brief screening tool to assist clinicians in identifying patients with CS-related sign and symptoms predominance but should not be used as a stand-alone tool.

Our findings indicate that clinical indicators are an accurate tool for the identification of the CS-related sign and symptoms. Although the majority of patients with impaired CPM presented CS-related sign and symptoms, there were 20% of participants who presented divergent results. Therefore, the clinical presentation of the CS-related sign and symptoms in patients with musculoskeletal pain may be disparate from the psychophysical test, which can reveal the neurophysiological impairment. The conflicting diagnosis of the CS-related sign and symptoms in the clinical presentation and the neurophysiological test preclude adequate decision making in patients with musculoskeletal pain. Additionally, the clinical indicators were developed to identify the predominance of pain in patients with low back pain [20]. Thus, patients with predominance of nociceptive or peripheral neuropathic pain may also present in the impairment of descending nociceptive inhibitory system. For instance, the results of the study of Fingleton et al. [37] demonstrated the presence of CS in patients with knee osteoarthritis, which is regularly considered nociceptive pain. Despite these limitations, our results highlight that patients without the predominance of the CS-related sign and symptoms do not present impairment of the CPM.

Few studies have investigated the validity of the clinical tools to identify patients with CS-related sign and symptoms. For instance, Gervais-Hupe et al. observed sensitivity of 87.2% and specificity of 34.2% in the identification of CS using the Central Sensitization Inventory (CSI) with a cut-point of 22 in patients with knee osteoarthritis when compared to CPT [38]. The same study showed that the painDETECT had a sensitivity of 61.5% and specificity of 77.6% in the identification of CS using a cut-point of 12 [38]. Thus, the CSI may represent an adequate instrument to identify patients with CS. In contrast, the clinical indicators and the painDETECT are appropriate tools to rule out those patients. Future studies should concentrate on methods to pragmatically characterization of patients with CS-related sign and symptoms to facilitate the decision making of the clinicians.

## Conclusion

Clinical indicators demonstrated a valuable tool for detecting the impaired CPM, which is a remarkable feature of the CS-related sign and symptoms. Clinicians are encouraged to use the clinical indicators in the management of patients with musculoskeletal pain.

## Data Availability

Not applicable.
